# Long-Term Air Pollution Exposure and Blood Pressure in the Sister Study

**DOI:** 10.1289/ehp.1408125

**Published:** 2015-03-06

**Authors:** Stephanie H. Chan, Victor C. Van Hee, Silas Bergen, Adam A. Szpiro, Lisa A. DeRoo, Stephanie J. London, Julian D. Marshall, Joel D. Kaufman, Dale P. Sandler

**Affiliations:** 1 Department of Environmental and Occupational Health Sciences, and; 2 Department of Medicine, University of Washington, Seattle, Washington, USA; 3 Department of Mathematics & Statistics, Winona State University, Winona, Minnesota, USA; 4 Department of Biostatistics, University of Washington, Seattle, Washington, USA; 5 Chronic Disease Epidemiology Group, and; 6 Genetics, Environment, & Respiratory Disease Group, National Institute of Environmental Health Sciences, National Institutes of Health, Department of Health and Human Services, Research Triangle Park, North Carolina, USA; 7 Department of Civil Engineering, University of Minnesota, Minneapolis, Minnesota, USA; 8 Department of Epidemiology, University of Washington, Seattle, Washington, USA

## Abstract

**Background:**

Exposure to air pollution has been consistently associated with cardiovascular morbidity and mortality, but mechanisms remain uncertain. Associations with blood pressure (BP) may help to explain the cardiovascular effects of air pollution.

**Objective:**

We examined the cross-sectional relationship between long-term (annual average) residential air pollution exposure and BP in the National Institute of Environmental Health Sciences’ Sister Study, a large U.S. cohort study investigating risk factors for breast cancer and other outcomes.

**Methods:**

This analysis included 43,629 women 35–76 years of age, enrolled 2003–2009, who had a sister with breast cancer. Geographic information systems contributed to satellite-based nitrogen dioxide (NO_2_) and fine particulate matter (≤ 2.5 μm; PM_2.5_) predictions at participant residences at study entry. Generalized additive models were used to examine the relationship between pollutants and measured BP at study entry, adjusting for cardiovascular disease risk factors and including thin plate splines for potential spatial confounding.

**Results:**

A 10-μg/m^3^ increase in PM_2.5_ was associated with 1.4-mmHg higher systolic BP (95% CI: 0.6, 2.3; *p* < 0.001), 1.0-mmHg higher pulse pressure (95% CI: 0.4, 1.7; *p* = 0.001), 0.8-mmHg higher mean arterial pressure (95% CI: 0.2, 1.4; *p* = 0.01), and no significant association with diastolic BP. A 10-ppb increase in NO_2_ was associated with a 0.4-mmHg (95% CI: 0.2, 0.6; *p* < 0.001) higher pulse pressure.

**Conclusions:**

Long-term PM_2.5_ and NO_2_ exposures were associated with higher blood pressure. On a population scale, such air pollution–related increases in blood pressure could, in part, account for the increases in cardiovascular disease morbidity and mortality seen in prior studies.

**Citation:**

Chan SH, Van Hee VC, Bergen S, Szpiro AA, DeRoo LA, London SJ, Marshall JD, Kaufman JD, Sandler DP. 2015. Long-term air pollution exposure and blood pressure in the Sister Study. Environ Health Perspect 123:951–958; http://dx.doi.org/10.1289/ehp.1408125

## Introduction

There is a well-established relationship between combustion-related air pollution exposure—especially particulate matter ≤ 2.5 μm in diameter (PM_2.5_)—and cardiovascular disease (CVD) morbidity and mortality (Brook et al. 2010). Although there have been numerous studies that demonstrate this relationship, the mechanisms are poorly understood.

One potential mechanism is an effect of inhaled air pollution on blood pressure (BP), mediated through autonomic nervous system dysfunction and/or changes in inflammation and oxidative stress. Increased BP is a strong risk factor for CVD including increases in left ventricular mass, which have been associated with long-term air pollution exposures (Van Hee et al. 2009).

Recent work has suggested that short-term (hours to days) particulate matter and traffic-related pollutant exposures may lead to transient increases in BP (Baccarelli et al. 2011; Baumgartner et al. 2011; Brook et al. 2011; Cosselman et al. 2012; Hoffmann et al. 2012; Langrish et al. 2012; Wu et al. 2013). In contrast, a study of 9,238 nonsmoking adults in Taiwan found reductions in systolic BP (SBP) and pulse pressure (PP) following short-term exposure to air pollution (Chen et al. 2012).

The relationship between chronic, long-term (e.g., yearly average) air pollution exposure and BP is less well understood, with some studies demonstrating an increase in BP associated with PM_2.5_ (Chuang et al. 2011; Fuks et al. 2011) and black carbon (Schwartz et al. 2012) exposure. Additional studies have investigated associations of BP with oxides of nitrogen (NO_x_; a marker of traffic-related pollution) (Dong et al. 2013; Sørensen et al. 2012), or have investigated the associations between BP and long-term exposures to both PM_2.5_ and gaseous traffic-related pollution exposure (Chuang et al. 2011; Coogan et al. 2012).

Developments in fine-scale spatial modeling of air pollution—using advanced statistical methods, geographic information systems, and both ground-based and satellite-based monitoring information—are now available. Together with large national cohorts, these exposure advances provide the opportunity for an improved analysis of this important research question.

We conducted a cross-sectional study to evaluate the relationship between BP (systolic, diastolic, pulse pressure, and mean arterial pressure) and long-term (annual average) exposure to PM_2.5_ and nitrogen dioxide (NO_2_) in a large U.S. cohort of women.

## Methods

*Study population*. Study participants were selected from the Sister Study, a large nationwide, prospective women’s cohort study investigating environmental and genetic risk factors for breast cancer and other diseases. 50,884 sisters of women with breast cancer, 35–76 years of age, were enrolled into the cohort between 2003 and 2009, as described elsewhere (Weinberg et al. 2007). The Sister Study was approved by the Institutional Review Board (IRB) of the National Institute of Environmental Health Sciences, National Institutes of Health, and the Copernicus Group IRB; all participants provided informed consent. In this analysis, participants were excluded due to residence outside of the continental United States (2% of participants), invalid address information (6%), missing BP measurement (0.3%), missing modeled NO_2_ estimates (0.06%), or other missing key covariate data (6%). Therefore, this analysis includes 43,629 (86%) of the recruited participants residing in the conterminous United States at enrollment.

Computer-assisted telephone interviews were administered by extensively trained staff, who collected information on participant demographics, socioeconomic status (SES) factors, residential history, occupational history, personal medical history (including self-reported diabetes, hypercholesterolemia, and hypertension), medication use, perceived stress (four-item perceived stress scale) (Cohen et al. 1983), and behavioral factors such as alcohol use and smoking. Participants were asked whether they had ever been diagnosed with diabetes, hypercholesterolemia, and hypertension by a medical professional. Responses were self-reported as no, yes, or “borderline,” with the last category added to accommodate participants who have been told that they had or nearly had the condition but did not require medications. Medication lists were coded using the Slone Drug Dictionary (Kelley et al. 2003), and anti-hypertensive medication use was defined as self-reporting one or more drugs in anti-hypertensive drug classes.

Women were enrolled throughout the United States and completed telephone interviews as close to the time they volunteered as possible; participation was not geographically or seasonally clustered. Home visits were conducted by examiners from a national company that performs insurance physicals, and were not scheduled in a manner to maximize geographic efficiency. The home visits provided measurements of anthropometry, fasting phlebotomy, and BP.

Approximately 10% of participants were sisters with one or more study participant, and the analyses do not account for familial clustering in the population because the most common cluster size was very small.

*Blood pressure ascertainment*. During baseline home visits, following consent and review of self-completed forms, participants were instructed to sit and rest for a few minutes before BP ascertainment. Trained examiners made three consecutive measurements of BP using an aneroid sphygmomanometer (model 760 & 775X; American Diagnostic Corporation). Measurements were taken from alternating arms, starting with the left arm using a left-right-left protocol, approximately 2 min apart. Examinations were scheduled, whenever possible, in the morning, and participants were encouraged to fast before the visit (excluding medications) and record whether anything had been taken by mouth.

For SBP and diastolic blood pressure (DBP) separately, the second and third measurements were averaged when three measurements were available. In some cases, examiners were unable to obtain three BP measurements. When only two BP measurements were available (*n* = 1,677), the two were averaged; and when only one BP measurement was recorded, the single value was used (*n* = 684).

Because the mechanism through which air pollution exposure may affect BP is not well understood, we also examined PP and mean arterial pressure (MAP), as other studies have also done (Auchincloss et al. 2008; Chen et al. 2012). PP, representing stroke volume and vascular compliance (Dart and Kingwell 2001), was determined by subtracting DBP from SBP; and MAP, a function of ventricular contractility, resistance, elasticity, and heart rate (Sesso et al. 2000), was calculated by PP/3 + DBP.

*Exposure assessment*. Participant home latitude and longitude at study entry was geocoded using ArcGIS 9.3.1 or 10.1 (ESRI, Redlands, CA) in conjunction with TeleAtlas Dynamap 2000 v16.1 road network (TeleAtlas, Boston, MA). Based on the residential geocodes, we assigned the census block.

For PM_2.5_, we developed a national prediction model for the year 2006, using partial least squares to select relevant components for the mean regression and universal kriging for spatial smoothing (Sampson et al. 2013). Briefly, the PM_2.5_ prediction model included satellite-based land use/land cover, road network characteristics, population density, vegetative index, distance to selected geographic features, and annual average U.S. Environmental Protection Agency’s Air Quality System monitor concentrations (http://www.epa.gov/ttn/airs/airsaqs/detaildata/downloadaqsdata.htm; see also Sampson et al. 2013). The model was fit using maximum likelihood, with each region having its own parameters (cross-validated *R*^2^ = 0.88). Individual PM_2.5_ concentrations were predicted for each residential geocode.

National NO_2_ predictions were developed using a previously described satellite-based land-use regression model for the year 2006 (Novotny et al. 2011). In short, atmospheric NO_2_ surface concentrations were predicted using multivariable linear regression based on land-use characteristics (impervious surfaces, tree canopy, sum of road lengths, elevation, and distance to coast) and tropospheric NO_2_ column abundance measurements from the Aura satellite’s ozone monitoring instrument (Novotny et al. 2011) (cross-validated *R*^2^ = 0.78). Individual NO_2_ concentrations were assigned based on the census block of the subject’s residential address.

Predicted annual average PM_2.5_ and NO_2_ concentrations were used to approximate long-term residential exposure at the time of baseline examination (2003–2009). The correlation between PM_2.5_ and NO_2_ for this population was 0.37, and although both exposure models contain similar terms, the modeling approaches are quite different.

*Other geographic covariate measurement*. To describe the overall urbanicity of the county in which participants reside, we used the Rural–Urban Continuum Codes of the U.S. Department of Agriculture (2013). The socioeconomic environment of the participants’ neighborhoods was defined by using neighborhood-level SES *z*-score based on U.S. Census block groups, which has been used in other studies (Diez Roux et al. 2001). A higher SES *z*-score signifies higher socioeconomic advantage.

*Statistical analysis*. For descriptive analyses, annual average air pollution exposure predictions (PM_2.5_ and NO_2_) and BP parameters (SBP, DBP, MAP, and PP) were divided into quartiles. Global *F*-tests (analysis of variance) were used to examine the differences in mean values of continuous variables (age, BP parameters, pollution measures) across quartiles of pollutants and BP parameters. The chi-square test was used to compare the frequencies of categorical variables across quartiles of exposure and outcomes. Categorical covariates were included in the main models and interactions as defined in Tables 1 and 2. To examine the overall spatial distribution of the exposures and outcomes, we plotted the mean BP parameters and air pollution exposure metrics for the participants by state, county, and census tract on U.S. maps.

**Table 1:** Baseline demographic characteristics of participants (*n*, mean ± SD, or %).

Characteristic	Quartile of exposure to PM_2.5_ (μg/m^3^)				Quartile of exposure to NO_2_ (ppb)				All participants
	2.2– 8.8	8.8– 10.8	10.8– 12.4	12.4– 17.4	1.0– 6.4	6.4– 9.2	9.2– 12.6	12.6– 34.2	
No. of participants (*n*)	10,929	10,924	10,915	10,861	10,927	10,917	10,884	10,901	43,629
Age (years)	55.5 ± 8.9	55.1 ± 9.1	54.8 ± 8.9	54.5 ± 8.9	55.3 ± 8.8	55.1 ± 8.9	54.8 ± 9.0	54.7 ± 9.0	55.0 ± 8.9
Race or ethnic group (%)
Non-Hispanic white	92	89	86	75	91	88	84	78	85
Black	2	5	9	20	5	7	10	14	9
Hispanic	3	3	3	3	1	2	4	5	3
Other	3	3	2	2	3	3	2	3	3
Household income (%)
< $20,000	26	25	23	25	28	25	23	23	25
$20,000 to < $50,000	45	45	44	43	46	45	44	41	44
$50,000 to < $100,000	26	26	28	27	23	25	28	30	27
≥ $100,000	4	4	5	5	3	4	4	6	4
Education (%)
≤ High school	14	16	15	14	17	16	14	12	15
Some college	35	35	32	32	37	35	33	31	34
Bachelor’s or above	51	49	52	53	46	50	53	57	52
Married (%)	76	72	72	63	80	74	68	60	71
Working > 20 hrs/week (%)	58	60	61	64	59	60	61	64	61
Perceived stress score (%)
Low (0–2)	60	57	57	55	59	58	57	55	57
Medium (3–6)	34	35	36	36	34	35	35	37	35
High (> 6)	7	8	8	8	7	7	8	8	8
Stable residence (%)	57	59	62	62	59	57	59	65	60
Neighborhood SES *z*-score tertile (%)
Low	31	34	31	37	45	32	27	30	33
Medium	37	34	32	30	35	36	33	29	33
High	31	32	37	33	20	33	39	41	33
Rural–Urban Continuum Code (%)
Metro area ≥ 1 million	39	58	58	72	25	44	67	90	57
Metro area < 1 million	39	29	31	23	42	41	30	10	31
Non-metro county	22	12	12	5	33	14	3	0	13
Metro, metropolitan. Shown as annual neighborhood SES *z*-score tertile: The socioeconomic environment of the participants’ neighborhoods was defined by U.S. Census block group characteristics. A higher SES *z*-score signifies higher socioeconomic advantage.									

**Table 2:** Baseline health characteristics of participants (mean ± SD or %).

Characteristic	Quartile of exposure to PM_2.5_ (μg/m^3^)				Quartile of exposure to NO_2_ (ppb)				All participants
	2.2–8.8	8.8–10.8	10.8–12.4	12.4–17.4	1.0–6.4	6.4–9.2	9.2–12.6	12.6–34.2	
Systolic BP (mmHg)	114.3 ± 13.7	114.6 ± 13.2	114.8 ± 13.6	115.6 ± 14.1	115.2 ± 13.5	114.6 ± 13.6	114.4 ± 13.6	115.0 ± 13.9	114.8 ± 13.6
Diastolic BP (mmHg)	72.0 ± 8.8	72.2 ± 8.6	72.3 ± 8.7	73.1 ± 9	72.5 ± 8.6	72.3 ± 8.8	72.2 ± 8.9	72.5 ± 8.9	72.4 ± 8.8
Mean arterial (mmHg)	86.1 ± 9.7	86.3 ± 9.3	86.4 ± 9.5	87.3 ± 9.9	86.8 ± 9.4	86.4 ± 9.6	86.3 ± 9.7	86.7 ± 9.7	86.5 ± 9.6
Pulse pressure (mmHg)	42.3 ± 9.7	42.4 ± 9.6	42.5 ± 9.9	42.4 ± 10	42.7 ± 9.8	42.3 ± 9.8	42.2 ± 9.7	42.6 ± 9.9	42.4 ± 9.8
Body mass index (kg/m^2^)									
Normal (< 25)	42	39	38	34	37	38	39	39	38
Overweight (25 to < 30)	31	31	32	32	33	32	32	31	32
Obese (≥ 30)	27	30	30	34	30	30	30	30	30
Smoking status									
Never	53	53	53	55	54	55	54	51	53
Former	40	38	38	36	37	37	38	40	38
Current	7	8	8	9	9	8	8	9	8
Alcohol use									
Never	3	3	3	4	4	3	3	3	3
Former	14	15	15	16	16	15	14	14	15
Current	84	82	82	80	80	82	83	83	82
Diabetes									
Yes	5	6	6	7	6	5	6	6	6
No	93	92	92	90	91	92	91	91	92
Borderline	3	3	3	3	3	3	3	3	3
Hypercholesterolemia									
Yes	32	34	33	33	34	33	33	32	33
No	56	54	55	55	54	55	56	56	55
Borderline	12	12	12	12	12	12	11	12	12
On BP medication	28	30	30	33	31	30	30	30	30
Hypertension									
Yes	25	27	27	30	27	27	28	27	27
No	71	69	69	65	68	68	69	69	69
Borderline	4	4	4	5	5	4	4	5	4
Borderline, self-reported classification that the participant had or nearly had the condition but did not require medications.									

We then fit multivariable linear models to investigate the relationship between individual BP parameters and each of the two pollutants of interest, adjusted for potential confounders including space [using unpenalized thin-plate regression splines (TPRS) in the MGCV package] (Wood 2003). TPRS are a flexible way of adjusting for spatial confounding. Using singular value decomposition, they decompose the distance matrix of all participant locations into a set of basis functions, the first *k* of which are included as adjustment covariates in the health models (Wood 2003).

Our final model included all covariates considered *a priori* as potential confounders. The *a priori* selection was based on a review of the literature before the analysis to avoid model selection bias. To evaluate the effect of groups of covariates, we added variables to successive models in series, with model 1 including age and race/ethnicity; model 2 also including SES variables (household income, education, marital status, working ≥ 20 hr per week outside the home, perceived stress score, and SES *z*-score); model 3 additionally including spatial features that are likely to vary both with pollution and BP (Rural–Urban Continuum code and TPRS for latitude and longitude); model 4 additionally including CVD risk factors [body mass index (BMI), waist-to-hip ratio, smoking status, alcohol use, history of diabetes, and history of hypercholesterolemia]; and the full model 5 additionally including BP medication use. For the categorical SES variables in model 2, we assume that collinearity does not exist because within the levels of each categorical variable there is some heterogeneity of the other categorical variables. Unpenalized TPRS for latitude and longitude were fit in two dimensions using 10 degrees of freedom (df). Statistical analyses were carried out using R 2.15.0 (R Core Team 2013) and Stata/IC 12.1 (StataCorp LP, College Station, TX). In all instances, a *p*-value of < 0.05 was considered significant.

When we observed significant associations with exposure in the full model, we additionally explored interactions with race/ethnicity, age, BMI, smoking, diabetes, and anti-hypertensive medication use by adding product terms of these variables with the exposure variable, and we examined interactive effect sizes and 95% confidence intervals (CIs) within strata using linear combinations of terms from the regression models (using wald.test and svycontrast in R).

Because there may be spatially varying characteristics that we were unable to account for, sensitivity analyses included varying the number of df for spatial adjustment and investigating the impact on main effect sizes and standard errors of alternate forms of the other independent and dependent variables (including nonlinear associations for the exposure metrics using penalized TPRS). To provide a complementary view, logistic regression was used to examine the hypertension as an outcome, defined as using anti-hypertensive medication or having an SBP ≥ 140 mmHg and DBP ≥ 90 mmHg. We also examined the effect of several subgroup analyses, restricting the full model analysis to individuals with stable residence (defined as the current address at the time of the examination representing their longest lived address) to account for potential exposure misclassification from characterizing current residence as a location of long-term exposure, and, separately, restricting the analysis to those with three valid, left-right-left arm, BP measurements to examine precision based on potential BP measurement error. Finally, we examined models including both air pollution exposure variables in a co-pollutant model.

## Results

*Participant characteristics*. Table 1 presents baseline demographic characteristics and Table 2 shows baseline health characteristics of participants, overall and by quartile of pollutant exposure. Among the 43,629 women, the mean ± SD age was 55 ± 8.9 years; range, 35–76 years. Thirty-one percent had self-reported hypertension or “borderline” hypertension, and 30% were on anti-hypertensive medications. Participants lived at their current address for a median of 11 years [interquartile range (IQR) of 16 years], ranging from < 1 year to 75 years.

*Bivariate associations*. Compared with the remainder of the sample, the highest quartile of both NO_2_ and PM_2.5_ exposure was significantly associated with younger participants, fewer non-Hispanic whites and more blacks, higher household income, fewer married women, more working > 20 hr/week, higher stress scores, greater residential stability, and with living in large metropolitan areas. Higher NO_2_ (but not PM_2.5_) quartile was associated with higher neighborhood SES, less overweightness, more former smokers, and more current alcohol users, whereas higher PM_2.5_ (but not NO_2_) was associated with significantly lower SES *z*-scores, more obesity, more current smokers, and fewer current alcohol users. NO_2_ was not associated with diabetes or anti-hypertensive medication use but was associated with self-reported hypertension and hypercholesterolemia, whereas higher PM_2.5_ was associated with more diabetes, higher anti-hypertensive use, and more self-reported hypertension but not hypercholesterolemia in these unadjusted univariate comparisons. All risk factors and other SES and geographic covariates were highly statistically significantly associated with quartiles of SBP, DBP, MAP, and PP (data not shown).

**Figure 1:**
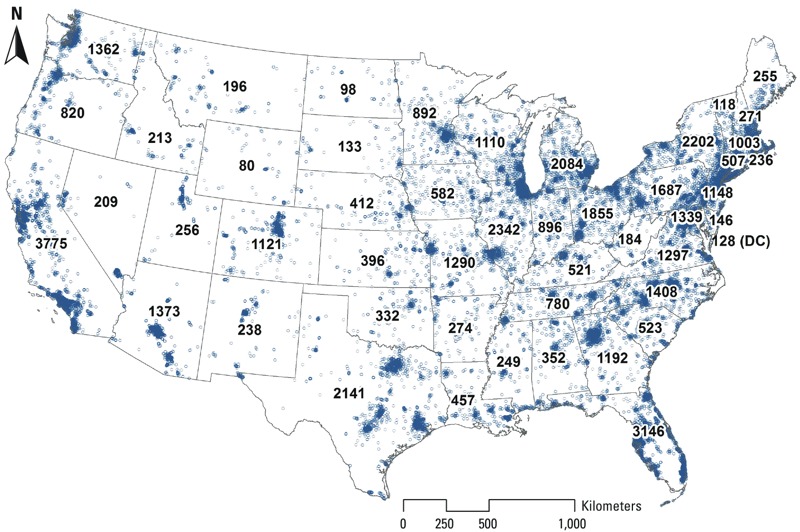
United States map of participant residential locations, with number of participants per state. Each participant is represented by an open blue circle.

**Figure 2:**
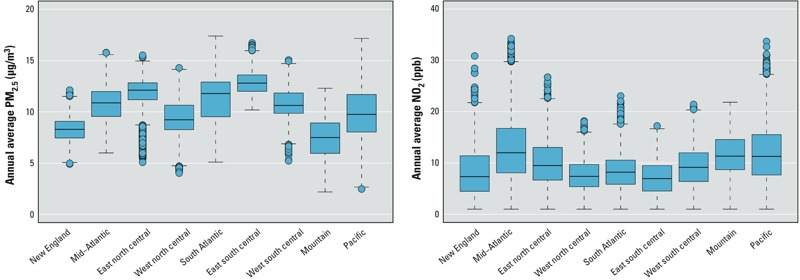
Boxplots of PM_2.5_ and NO_2_ participant annual average residential concentrations by U.S. census division. Boxes extend from the 25th to the 75th percentile, horizontal bars represent the median, whiskers extend 1.5 times the length of the interquartile range (IQR) above and below the 75th and 25th percentiles, respectively, and outliers are represented as points.

*Residential pollutant exposures*. Figure 1 shows the distribution of participants’ geocoded residential locations, with numbers representing the number of participants per state. The distribution of participants generally corresponds to the distribution of population across the United States. Figure 2 presents boxplots of the distribution of exposure predictions for PM_2.5_ and NO_2_, by U.S. census division. See Supplemental Material, Figures S1 and S2, for maps of mean pollutant levels of participants by U.S. census tract. PM_2.5_ shows large-scale spatial structure across the United States. NO_2_ exhibits a different spatial pattern, with high levels in highly urbanized areas, reflecting the traffic-related nature of NO_2_. Thus, PM_2.5_ exhibits greater between-city variability, whereas NO_2_ exhibits more within-city variability.

**Figure 3:**
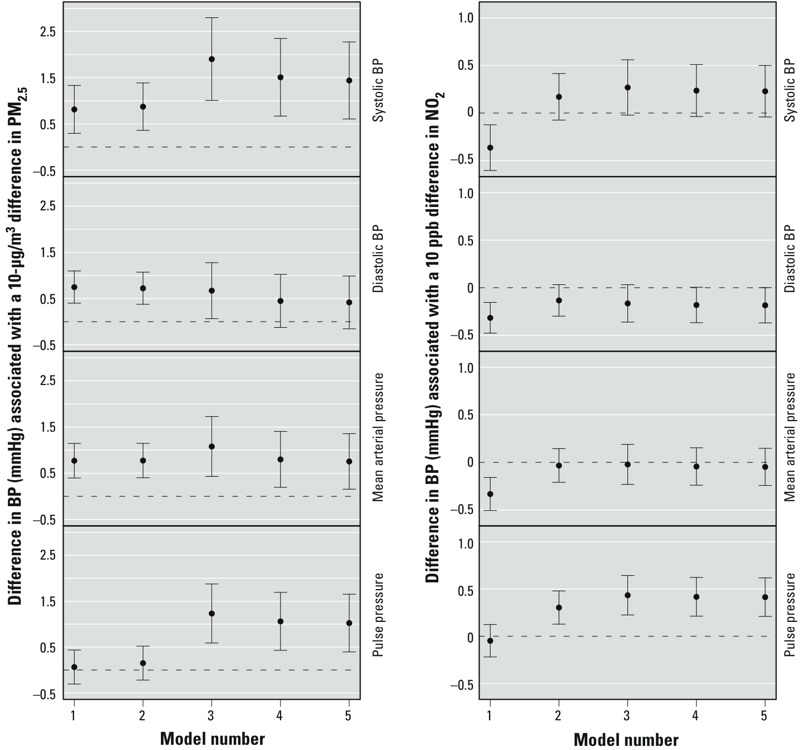
Relationship between blood pressure and annual average air pollution exposure for PM_2.5_ (left) and NO_2_ (right). Model 1: Included age and race/ethnicity. Model 2: model 1 + household income, education, marital status, working ≥ 20 hr per week outside the home, perceived stress score, and socioeconomic status *z*-score. Model 3: model 2 + Rural–Urban Continuum Codes and unpenalized thin-plate regression splines for latitude and longitude. Model 4: model 3 + body mass index, waist-to-hip ratio, smoking status, alcohol use, history of diabetes, and history of hypercholesterolemia. Model 5: model 4 + blood pressure medication use.

*Adjusted relationship between pollutants and BP*. Figure 3 shows the results of adjusted linear models by pollutant. In the fully adjusted models (model 5) shown in Table 3, a 10-μg/m^3^ increment in PM_2.5_ was associated with a 1.4-mmHg higher SBP (95% CI: 0.6, 2.3; *p* < 0.001), a 1.0-mmHg higher PP (95% CI: 0.4, 1.7; *p* = 0.001), a 0.8-mmHg higher MAP (95% CI: 0.2, 1.4; *p* = 0.01), and a 0.4-mmHg higher DBP (95% CI: –0.2, 1.0; *p* = 0.15). A 10-ppb increase in NO_2_ was associated with a 0.4-mmHg (95% CI: 0.2, 0.6; *p* < 0.001) higher PP, a 0.2-mmHg higher SBP (95% CI: 0.0, 0.5; *p* = 0.10), a 0.2-mmHg lower DBP (95% CI: –0.4, 0.0; *p* = 0.05), and no difference in MAP (95% CI: –0.2, 0.1; *p* = 0.63).

**Table 3:** Estimated effect of PM_2.5_ and NO_2_ exposure on blood pressure (mmHg), estimate (95% CI).

Outcome	Per 10 μg/m^3^ PM_2.5_ exposure		Per 10 ppb NO_2_ exposure	
	mmHg (95% CI)	*p*-Value	mmHg (95% CI)	*p*-Value
Systolic blood pressure
Model 1	0.8 (0.3, 1.3)	0.002	–0.4 (–0.6, –0.1)	0.003
Model 2	0.9 (0.4, 1.4)	< 0.001	0.2 (–0.1, 0.4)	0.17
Model 3	1.9 (1.0, 2.8)	< 0.001	0.3 (0.0, 0.6)	0.07
Model 4	1.5 (0.7, 2.4)	< 0.001	0.2 (0.0, 0.5)	0.09
Model 5	1.4 (0.6, 2.3)	< 0.001	0.2 (0.0, 0.5)	0.10
Diastolic blood pressure
Model 1	0.8 (0.4, 1.1)	< 0.001	–0.3 (–0.5, –0.2)	< 0.001
Model 2	0.7 (0.4, 1.1)	< 0.001	–0.1 (–0.3, 0.0)	0.11
Model 3	0.7 (0.1, 1.3)	0.03	–0.2 (–0.4, 0.0)	0.10
Model 4	0.5 (–0.1, 1.0)	0.12	–0.2 (–0.4, 0.0)	0.06
Model 5	0.4 (–0.2, 1.0)	0.15	–0.2 (–0.4, 0.0)	0.05
Mean arterial pressure
Model 1	0.8 (0.4, 1.2)	< 0.001	–0.3 (–0.5, –0.2)	< 0.001
Model 2	0.8 (0.4, 1.2)	< 0.001	0.0 (–0.2, 0.1)	0.72
Model 3	1.1 (0.4, 1.7)	0.001	0.0 (–0.2, 0.2)	0.84
Model 4	0.8 (0.2, 1.4)	0.01	0.0 (–0.2, 0.2)	0.67
Model 5	0.8 (0.2, 1.4)	0.01	–0.1 (–0.2, 0.2)	0.63
Pulse pressure
Model 1	0.1 (–0.3, 0.4)	0.73	–0.1 (–0.2, 0.1)	0.59
Model 2	0.2 (–0.2, 0.5)	0.42	0.3 (0.1, 0.5)	< 0.001
Model 3	1.2 (0.6, 1.9)	< 0.001	0.4 (0.2, 0.6)	< 0.001
Model 4	1.1 (0.4, 1.7)	< 0.001	0.4 (0.2, 0.6)	< 0.001
Model 5	1.0 (0.4, 1.7)	0.001	0.4 (0.2, 0.6)	< 0.001
Model 1: Included age and race/ethnicity. Model 2: Model 1 + household income, education, marital status, working ≥ 20 hr per week outside the home, perceived stress score, and socioeconomic status *z*-score. Model 3: Model 2 + Rural-Urban Continuum Codes and unpenalized thin-plate regression splines for latitude and longitude. Model 4: Model 3 + body mass index, waist-to-hip ratio, smoking status, alcohol use, history of diabetes, and history of hypercholesterolemia. Model 5: Model 4 + blood pressure medication use.				

For PM_2.5_, adjustment for spatial features (model 3 vs. model 2) had the largest impact on effect estimates reflecting the large-scale spatial structure in PM_2.5_, with an increase in the positive association with SBP, a slight decrease in the positive association with DBP, and a concomitant increase in the PP association after adjustment (Table 3). For NO_2_, adjustment for variables representing individual and neighborhood SES (model 2 vs. model 1) had the largest impact on effect estimates particularly for SBP, with the association changing from negative and statistically significant to positive and approaching statistical significance. The importance of adjusting for these variables reflects the within-city nature of NO_2_ variability. After full adjustment, associations with NO_2_ and SBP are positive and DBP are negative, leading to a significant positive association with total PP. In general, all other added potentially confounding variables showed little impact on effect estimates.

*Interactions*. For our finding of an association between PM_2.5_ and SBP, there was no significant evidence of interaction with BMI, race/ethnicity, age, smoking, diabetes, or anti-hypertensive medication use (see Supplemental Material, Figure S3).

*Sensitivity analyses*. The results of varying the number of df used for spatial adjustment are shown in Supplemental Material, Figures S4 and S5. For PM_2.5_ the estimated associations with BP were fairly stable with ≥ 8 df. Varying the df had little impact on the associations of BP with NO_2_. Using natural logarithmic transformations of the exposure and outcome variables produced no appreciable changes in the overall findings of the analysis (data not shown). When the analysis was restricted to participants with residential stability (*n* = 26,217), PM_2.5_ effect estimates for SBP and PP were somewhat stronger; a 10-μg/m^3^ increase in PM_2.5_ was associated with a 2.1-mmHg higher SBP (95% CI: 1.0, 3.2; *p* < 0.001) and a 1.6-mmHg higher PP (95% CI: 0.7, 2.4; *p* < 0.001), and no substantive changes in other effect estimates (data not shown). Restricting the analysis to participants with three valid BP measurements at the examination (*n* = 41,263) also produced no change in estimates (data not shown).

The results of sensitivity analyses using penalized TPRS to assess nonlinearity of associations between BP and the exposures of interest were generally consistent with linearity, with some evidence of nonlinearity at the extremes of the exposure distributions (data not shown).

Neither a 10-μg/m^3^ increase in PM_2.5_ nor a 10-ppb increase in NO_2_ exposure was associated with increased odds of hypertension in model 5 (OR: 0.95, 95% CI: 0.38, 2.36, *p* = 0.92; OR: 1.02, 95% CI: 0.75, 1.38, *p* = 0.91, respectively).

Though not observed for SBP, PP, or MAP, we saw a quadratic association between DBP and age. Using a quadratic rather than linear adjustment for age in the DBP models yielded null results between DBP and both exposures (data not shown). Age range did not vary across quartiles of exposure (data not shown).

*Co-pollutant analysis*. Results from the co-pollutant analysis are shown in Supplement Material, Table S1. In the models that included both NO_2_ and PM_2.5_, the positive association between PM_2.5_ and DBP became stronger and statistically significant whereas the association with PP became essentially null and insignificant. Specifically in fully adjusted models (model 5), a 10-μg/m^3^ increase in PM_2.5_ was associated with a 1.2-mmHg higher DBP (95% CI: 0.5, 1.9; *p* = 0.001) and a 0.4-mmHg higher PP (95% CI: –0.4, 1.2; *p* = 0.3). The negative association between NO_2_ and DBP became stronger and remained statistically significant in the co-pollutant analysis, whereas the association between NO_2_ and MAP became stronger and statistically significant. For NO_2_, a 10-ppb increase in NO_2_ was associated with a 0.4-mmHg lower DBP (95% CI: –0.6, –0.2; *p* < 0.001) and a 0.3-mmHg lower MAP (95% CI: –0.5, –0.1; *p* = 0.02). No other associations were meaningfully changed from the primary single-pollutant models.

## Discussion

This is the first large national cohort studied with individual BP measurements and the use of advanced modeling methods to assess fine-scale intraurban gradients in major criteria air pollutants, PM_2.5_ and NO_2_. Prior studies have either used coarser-scale exposure assessment (e.g., nearest regulatory monitor) or administrative records (e.g., records of hypertension diagnoses) for outcome assessment. With exposures in the range currently experienced in the United States, these findings are interesting and important.

Our study demonstrates an association between increases in long-term residential exposure to PM_2.5_ and NO_2_ and higher measures of blood pressure (SBP, PP, and MAP for PM_2.5_ and PP for NO_2_). These relationships were robust to adjustment for multiple potential confounders, including SES and spatial characteristics, and apparently without threshold. The study also found an inverse relationship between NO_2_ and DBP in the fully adjusted model (model 5). We saw little evidence of effect modification by age, race/ethnicity, smoking, diabetes, anti-hypertensive medication use, or BMI (see Supplemental Material, Figure S3). Evidence of a long-term impact of air pollution on BP in our study population provides support to the hypothesis that air pollution induces autonomic dysfunction that may ultimately lead to vascular remodeling, increased BP, and atherosclerosis (Brook et al. 2010).

Although these associations are modest at the individual level, the potential public health consequences of population-level changes in BP of this magnitude are substantial (Whelton et al. 2002). The effect sizes estimated in this study are the same order of magnitude as other traditionally recommended behavioral health interventions (He and MacGregor 2004). Because air pollution exposure is experienced at a population level, even a small pro-hypertensive response to long-term air pollution exposures could contribute significantly to CVD.

In this analysis, neither PM_2.5_ nor NO_2_ exposure was associated with increased odds of hypertension, consistent with findings elsewhere (Chen et al. 2014; Foraster et al. 2014; Fuks et al. 2011); this null finding may be attributable to misclassification of hypertension cases (many cases are unrecognized) or regional differences in diagnosis and treatment.

Few studies have examined the relationship between long-term average exposure to both PM_2.5_ and NO_2_ and BP, and none have done so over a large, spatially dispersed population such as this one. Furthermore, the few studies that have examined PP and/or MAP as outcomes focused on short-term air pollution exposure (Auchincloss et al. 2008; Chen et al. 2012; Dvonch et al. 2009; Zanobetti et al. 2004). Long-term average PM_2.5_ was shown to be associated with increased arterial BP in a population-based cohort study (*n* = 4,291) in a single metropolitan area in western Germany (Fuks et al. 2011). In Taiwan, a study with large air pollution exposure contrasts (*n* = 1,023) and no ability to account for neighborhood-level confounding showed strong positive associations between BP and both annual average PM_2.5_ and NO_2_ (Chuang et al. 2011). A study in an Ontario cohort found an association between PM_2.5_ estimated using satellite-based methods and the incidence of a hypertension diagnosis in electronic medical records (Chen et al. 2014).

In contrast, a Danish population-based cohort study (*n* = 57,053) found a small reduction in SBP with long-term average NO_x_ exposure (Sørensen et al. 2012). A study of Chinese adults (*n* = 24,845) found no relationship between nearest monitor NO_2_ and BP, but did find small increases in SBP and DBP in men associated with changes in PM_10_, SO_2_, and O_3_ (Dong et al. 2013). The inverse relationship between NO_2_ and DBP found in this study has not been reported by others (Chuang et al. 2011; Dong et al. 2013; Foraster et al. 2014), but it is possible that the inverse results may have been related to residual confounding.

Alternatively, differences in exposure metrics (NO_x_ vs. NO_2_) or other modeling methods may have contributed to differences in findings among studies. In a study of 853 elderly men in the Veterans Administration Normative Aging Study (Schwartz et al. 2012), positive associations between traffic particles and BP were observed.

Primary strengths of this study include its large size, high-quality measurements of BP, detailed characterization of potential confounders including individual and neighborhood-level SES and spatial features, its large geographic extent, and the use of estimates of exposure to both PM_2.5_ and NO_2_.

The cross-sectional nature of this study is its primary limitation. The cohort consists only of women and, thus, results might not be generalizable to men. Given that the cohort is composed entirely of sisters of women with breast cancer, it might also not be representative of the general U.S. female population. The prevalence of hypertension in the study population (31%) is similar to that of U.S. women (31.7%; 95% CI: 29.9%, 33.5%) according to the 2005–2008 National Health and Nutrition Examination Survey (Centers for Disease Control and Prevention 2011). Mean SBP was slightly lower and DBP was slightly higher in the study population (115 mmHg and 72 mmHg, respectively) compared with women in the general U.S. population (121 mmHg and 70 mmHg, respectively) (Wright et al. 2011).

PM_2.5_ and NO_2_ exposures were modeled for the year 2006, whereas BP was measured between 2003 and 2009. The air pollution measures linked to residence at time of study enrollment were chosen as generally representative of long-term air pollution exposure. When our analysis was restricted to participants with residential stability, effect estimates appeared somewhat larger, suggesting that bias in these reported associations resulting from this exposure measurement error may underestimate the true associations.

The results may also have been affected by exposure misclassification. This study evaluated long-term residential air pollution exposure, and did not account for occupational, personal, or indoor air pollution exposure. There may be residual confounding by short-term exposure to air pollution that this study was unable to account for, which was associated with higher SBP and DBP in a study of young adults in Taiwan (Lin et al. 2009). Additionally, the analysis assessed the effects of a 10-μg/m^3^ change in PM_2.5_ (IQR, 3.58 μg/m^3^; 10th–90th percentile, 7.38–13.38 μg/m^3^) and a 10-ppb change in NO_2_ (IQR, 6.21 ppb; 10th–90th percentile, 4.11–16.41 ppb) which may be extrapolating beyond the data in some regions or comparing extremes of the exposure distributions. A moderate amount of correlation between PM_2.5_ and NO_2_ was observed (*R* = 0.37), suggesting that one exposure is not acting as a surrogate for the other, which is consistent with other studies that have reported differences in associations with BP based on multi-pollutant models compared with single-pollutant models (Chuang et al. 2011; Coogan et al. 2012).

Despite the detailed characterization of potential confounders, most were self-reported, including medication lists used to determine anti-hypertensive medication use. Similarly, physical activity and diet were not included, which could affect validity of the results via residual confounding; it is possible that the spatial adjustments may capture some of the anticipated variation in physical activity and diet. Although anti-hypertensive treatment lowers blood pressure, there was not an ideal way to account for medication use in our analysis; it does not appear to behave as a confounder in this analysis (Foraster et al. 2014).

BP ascertainment on a single day does not allow a precise measurement of the individual’s true BP levels. Whenever possible, BP was measured in the morning, but hour of measurement was not included in the analysis. Although seasonal trends in BP could contribute to nondifferential misclassification, no discernible patterns were observed when reviewing exam month by geographic region.

Potential residual confounding by traffic noise is a possibility (Dratva et al. 2012; Sørensen et al. 2011). However, confounding by noise in this study might be limited given the wide area studied and the large sample size, as demonstrated elsewhere (Tétreault et al. 2013).

## Conclusions

Our findings suggest that chronic PM_2.5_ exposure may lead to increases in both SBP and PP, and that chronic NO_2_ exposure may increase PP. These findings are consistent with our hypothesis that air pollution leads to CVD through mechanisms involving increased BP, potentially via the long-term vascular remodeling that accompanies chronic autonomic dysfunction or inflammation and oxidative stress.

## Supplemental Material

(1.9 MB) PDF
