# Supplementary material for: Long-Term Air Pollution Exposure and Blood Pressure in the Sister Study
Source: Environ Health Perspect. 2015 Mar 6;123(10):951–8. doi: 10.1289/ehp.1408125 (PMC4590742; doi:10.1289/ehp.1408125)
Supplement: (1.9 MB) PDF [file ehp.1408125.s001.acco.pdf]

Environ Health Perspect
doi: 10.1289/ehp.1408125

Note to Readers: ***EHP*** **strives to ensure that all journal content is accessible to all readers. However, some figures and Supplemental Material published in** ***EHP*** **articles may not conform to 508 standards due to the complexity of the information being presented. If you need assistance accessing journal content, please contact ehp508@niehs.nih.gov. Our staff will work with you to assess and meet your accessibility needs within 3 working days.**

Supplemental Material

Long-Term Air Pollution Exposure and Blood Pressure in the Sister Study

Stephanie H. Chan, Victor C. Van Hee, Silas Bergen, Adam A. Szpiro, Lisa A. DeRoo, Stephanie J. London, Julian D. Marshall, Joel D. Kaufman, and Dale P. Sandler

Table of Contents

Figure S1. Mean participant $PM_{2.5}$ concentration. Based on residential location at the time of baseline examination, long-term fine particulate matter ($PM_{2.5}$) exposure was predicted for each participant for the year 2006 in μg/m^3 (Sampson et al. 2013). The map shows the mean concentration of $PM_{2.5}$ for U.S. census tracts with participants.

Figure S2. Mean participant NO_2 concentration. Based on residential location at the time of baseline examination, long-term nitrogen dioxide (NO_2) exposure was predicted for each participant's U.S. census block for the year 2006 in ppb (Novotny et al. 2011). The map shows the mean concentration of NO_2 for U.S. census tracts with participants.

Figure S3. Visualization of interactions for SBP and $PM_{2.5}$ for categorical variables. Circles represent stratum-specific estimates between $PM_{2.5}$ and SBP adjusting for all other Model 5 covariates as main effects with 95% confidence intervals.

Figure S4. Varying degrees of freedom for spatial adjustment with $PM_{2.5}$. The primary analysis used 10 degrees of freedom, which was varied from 6 to 12 in sensitivity analyses. The fully adjusted model included age, race/ethnicity, household income, education, marital status,

working more than 20 hours per week outside the home, perceived stress score, neighborhood socioeconomic status Z score, urban-rural continuum code, thin plate splines for latitude and longitude, body mass index, waist-to-hip ratio, smoking status, alcohol use, diabetes, hypercholesterolemia, and blood pressure medication use.

Figure S5. Varying degrees of freedom for spatial adjustment with NO_2. The primary analysis used 10 degrees of freedom, which was varied from 6 to 12 in sensitivity analyses. The fully adjusted model included age, race/ethnicity, household income, education, marital status, working more than 20 hours per week outside the home, perceived stress score, neighborhood socioeconomic status Z score, urban-rural continuum code, thin plate splines for latitude and longitude, body mass index, waist-to-hip ratio, smoking status, alcohol use, diabetes, hypercholesterolemia, and blood pressure medication use.

Table S1. Co-pollutant models and blood pressure (mmHg), estimate (95% CI).

References

Figure S1. Mean participant $PM_{2.5}$ concentration**.** Based on residential location at the time of baseline examination, long-term fine particulate matter ($PM_{2.5}$) exposure was predicted for each participant for the year 2006 in μg/m^3 (Sampson et al. 2013). The map shows the mean concentration of $PM_{2.5}$ for U.S. census tracts with participants.

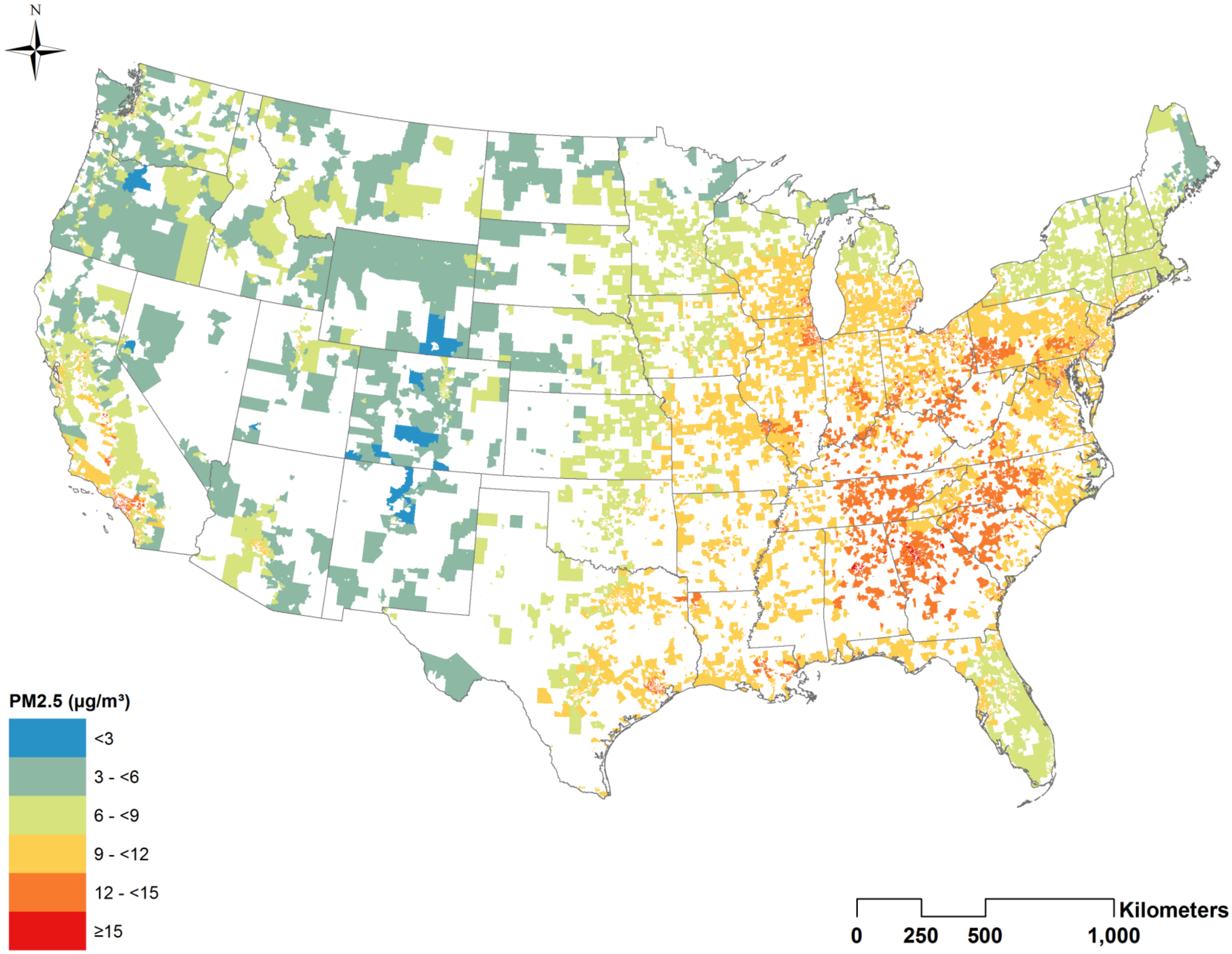

Figure S2. Mean participant NO_2 concentration. Based on residential location at the time of baseline examination, long-term nitrogen dioxide (NO_2) exposure was predicted for each participant's U.S. census block for the year 2006 in ppb (Novotny et al. 2011). The map shows the mean concentration of NO_2 for U.S. census tracts with participants.

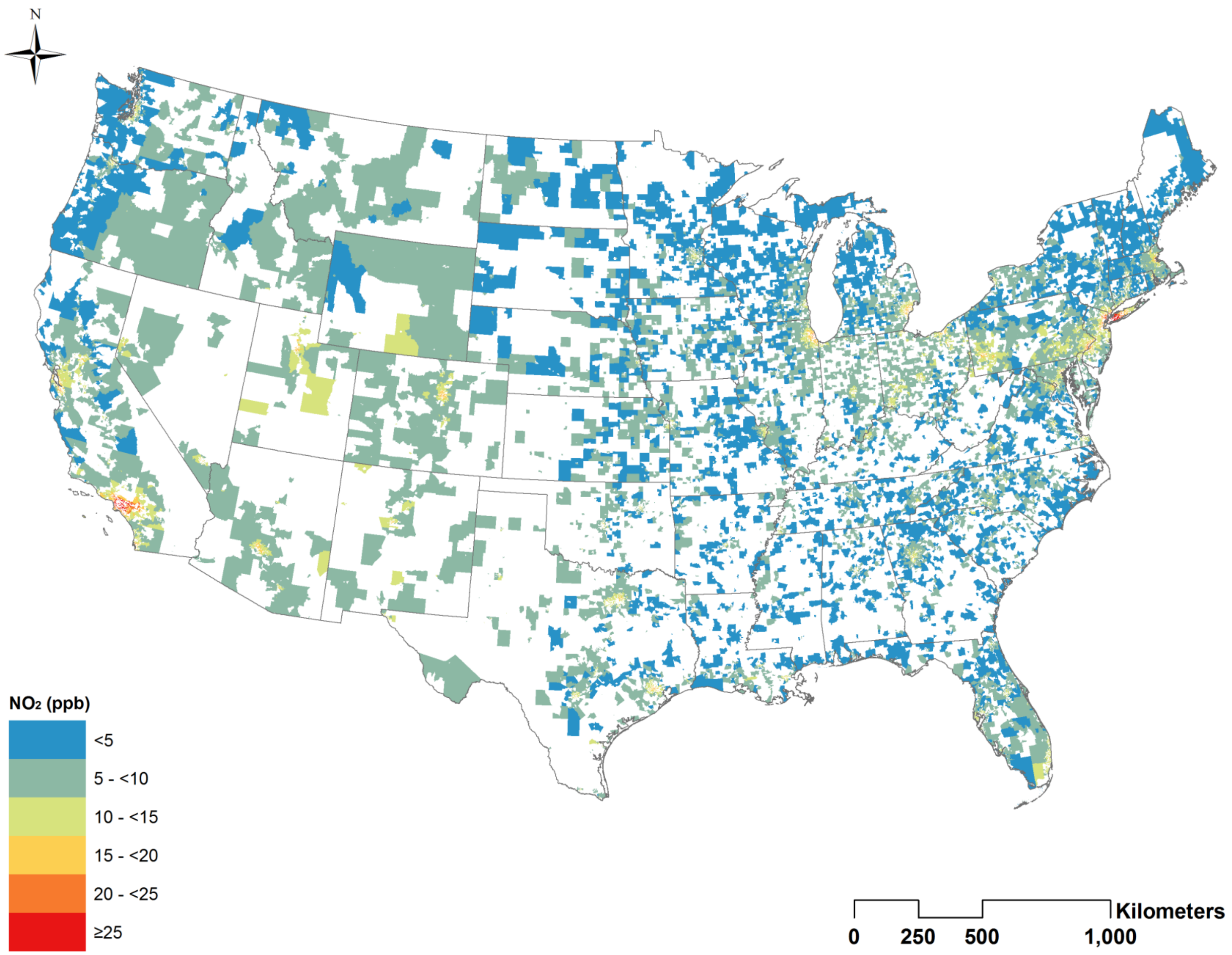

Figure S3. Visualization of interactions for SBP and $PM_{2.5}$ for categorical variables. Circles represent stratum-specific estimates between $PM_{2.5}$ and SBP adjusting for all other Model 5 covariates as main effects with 95% confidence intervals.

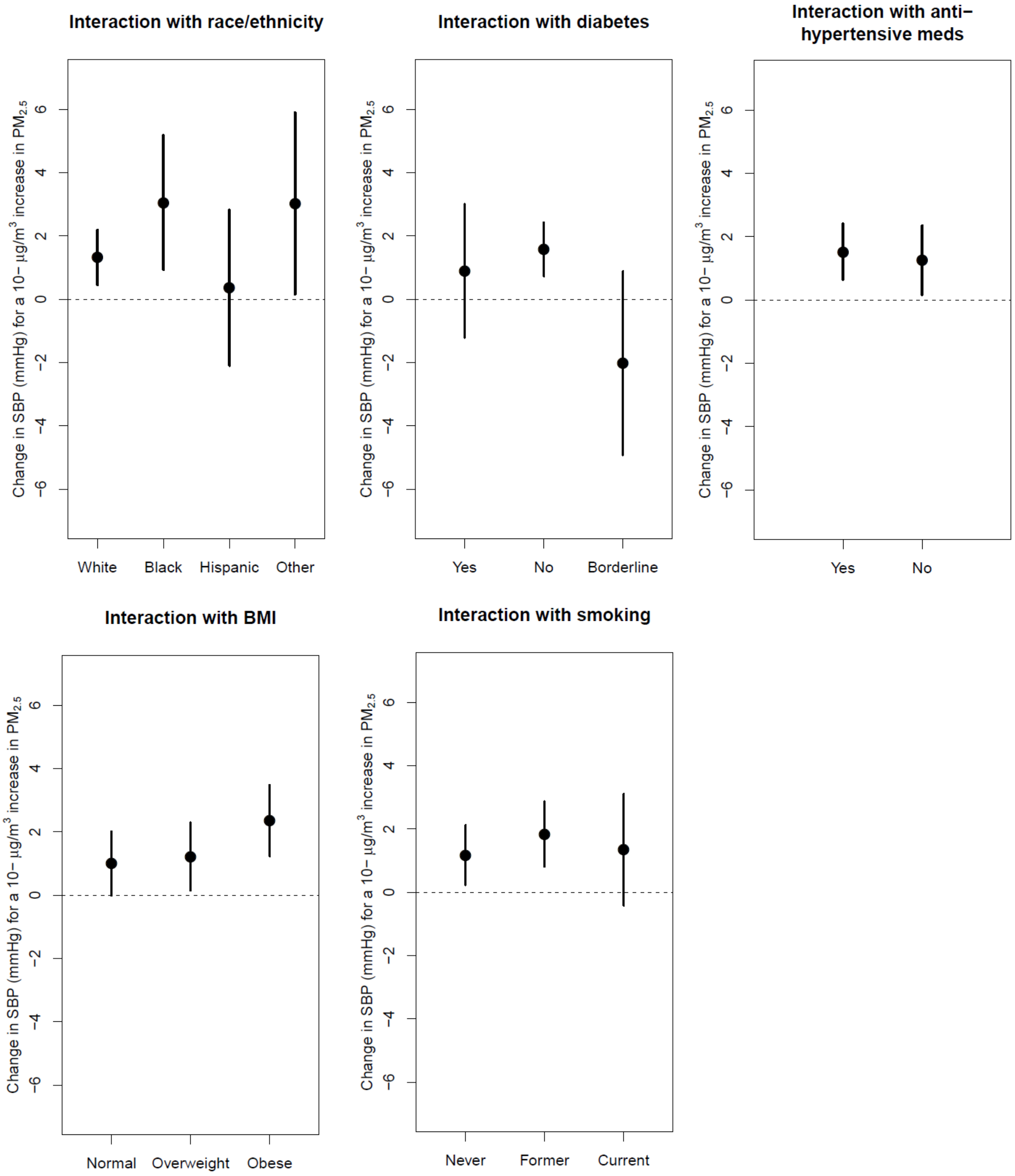

Figure S4. Varying degrees of freedom for spatial adjustment with $PM_{2.5}$. The primary analysis used 10 degrees of freedom, which was varied from 6 to 12 in sensitivity analyses. The fully adjusted model included age, race/ethnicity, household income, education, marital status, working more than 20 hours per week outside the home, perceived stress score, neighborhood socioeconomic status Z score, urban-rural continuum code, thin plate splines for latitude and longitude, body mass index, waist-to-hip ratio, smoking status, alcohol use, diabetes, hypercholesterolemia, and blood pressure medication use.

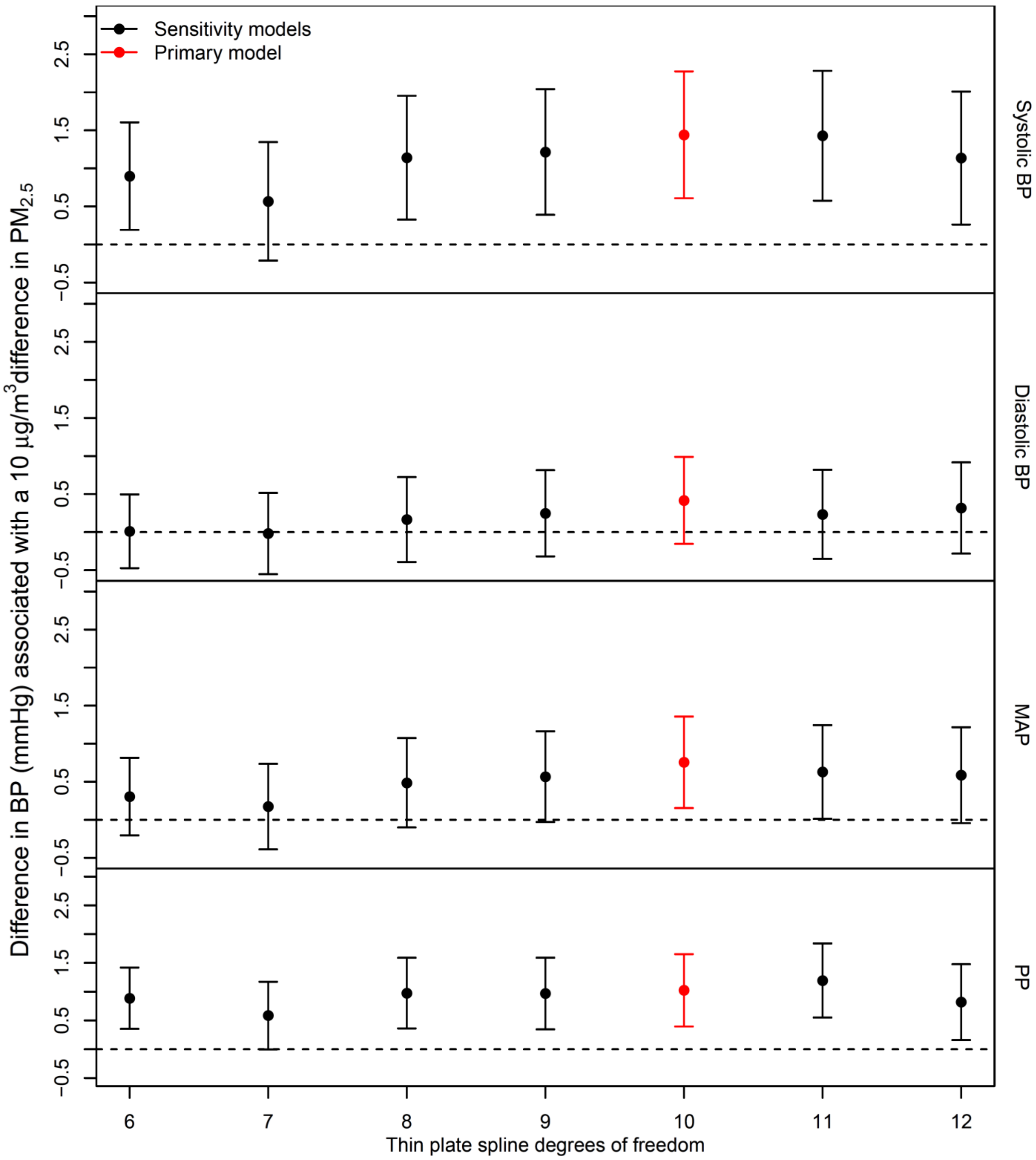

Figure S5. Varying degrees of freedom for spatial adjustment with NO_2. The primary analysis used 10 degrees of freedom, which was varied from 6 to 12 in sensitivity analyses. The fully adjusted model included age, race/ethnicity, household income, education, marital status, working more than 20 hours per week outside the home, perceived stress score, neighborhood socioeconomic status Z score, urban-rural continuum code, thin plate splines for latitude and longitude, body mass index, waist-to-hip ratio, smoking status, alcohol use, diabetes, hypercholesterolemia, and blood pressure medication use.

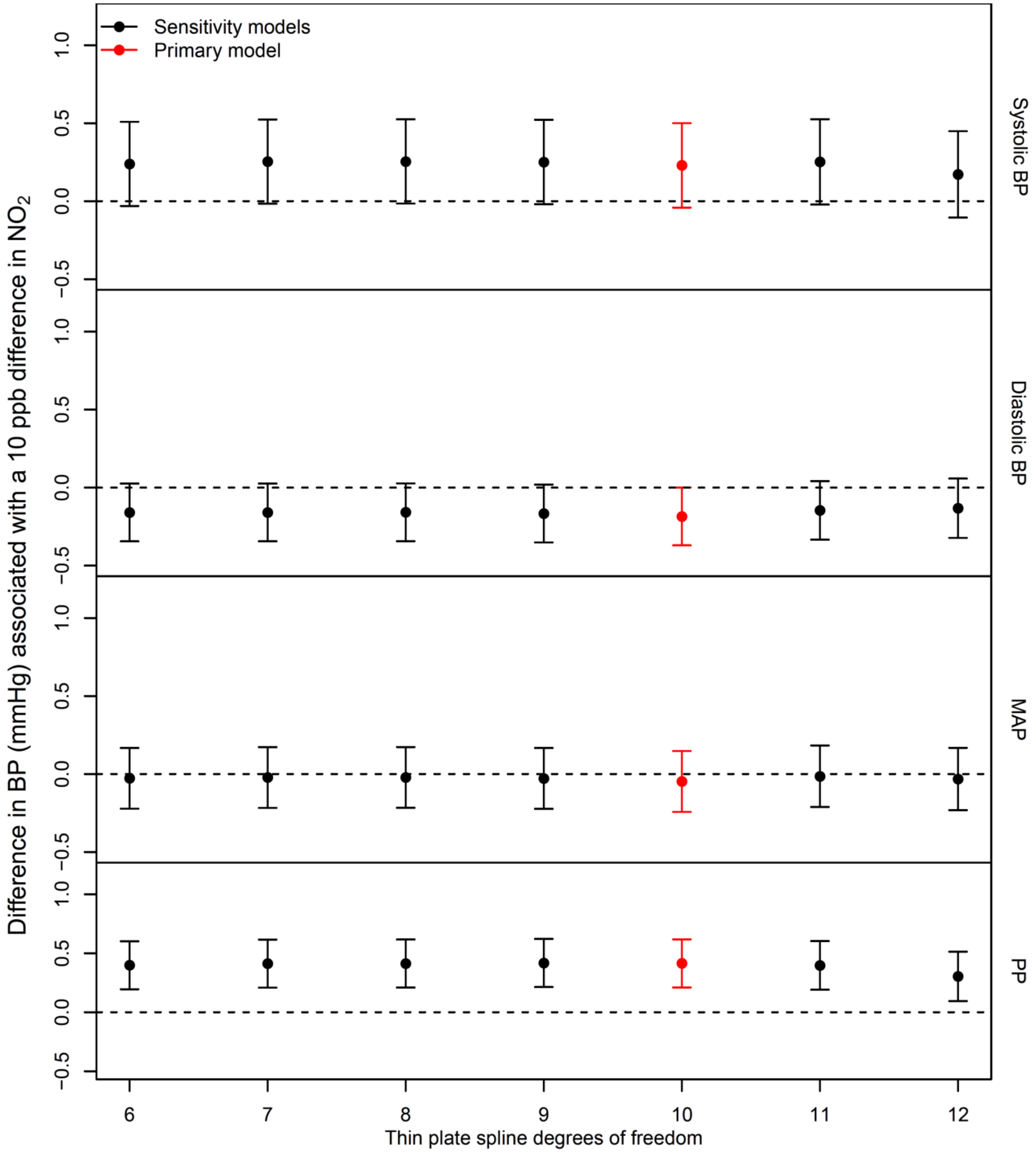

Table S1. Co-pollutant models and blood pressure (mmHg), estimate (95% CI).

Outcome	Per 10 μg/m^3 $PM_{2.5}$		Per 10 ppb NO_2	
Systolic Blood Pressure	1.6 (0.5, 2.6)	$p < 0.001$	-0.1 (-0.4, 0.3)	$p = 0.65$
Diastolic Blood Pressure	1.2 (0.5, 1.9)	$p < 0.001$	-0.4 (-0.6, -0.2)	$p < 0.001$
Mean Arterial Pressure	1.3 (0.6, 2.1)	$p < 0.001$	-0.3 (-0.5, -0.1)	$p = 0.02$
Pulse Pressure	0.4 (-0.4, 1.2)	$p = 0.31$	0.3 (0.1, 0.6)	$p = 0.01$

Fully adjusted for age, race/ethnicity, household income, education, marital status, working more than 20 hours per week outside the home, perceived stress score, neighborhood socioeconomic status Z score, urban-rural continuum code, thin plate splines for latitude and longitude, body mass index, waist-to-hip ratio, smoking status, alcohol use, diabetes, hypercholesterolemia, and blood pressure medication use.

References

Novotny EV, Bechle MJ, Millet DB, Marshall JD. 2011. National satellite-based land-use regression: NO2 in the United States. Environ Sci Technol 45:4407-14.

Sampson PD, Richards M, Szpiro AA, Bergen S, Sheppard L, Larson TV, et al. 2013. A regionalized national universal kriging model using Partial Lease Squares regression for estimating annual $PM_{2.5}$ concentrations in epidemiology. Atmospheric Environment 75:383-392.
